# White Lupin Adaptation to Moderately Calcareous Soils: Phenotypic Variation and Genome-Enabled Prediction

**DOI:** 10.3390/plants12051139

**Published:** 2023-03-02

**Authors:** Paolo Annicchiarico, Abco J. de Buck, Dimitrios N. Vlachostergios, Dennis Heupink, Avraam Koskosidis, Nelson Nazzicari, Margherita Crosta

**Affiliations:** 1 Research Centre for Animal Production and Aquaculture, Council for Agricultural Research and Economics, 26900 Lodi, Italy; 2 Luis Bolk Instituut, 3981 Bunnik, The Netherlands; 3 Institute of Industrial and Forage Crops, Hellenic Agricultural Organization “Demeter”, 41335 Larissa, Greece

**Keywords:** crop yield, genomic selection, GWAS, lime tolerance, *Lupinus albus*, plant-soil interaction

## Abstract

White lupin is a promising high-protein crop, the cultivation of which is limited by a lack of adaptation to soils that are even just mildly calcareous. This study aimed to assess the phenotypic variation, the trait architecture based on a GWAS, and the predictive ability of genome-enabled models for grain yield and contributing traits of a genetically-broad population of 140 lines grown in an autumn-sown environment of Greece (Larissa) and a spring-sown environment of the Netherlands (Ens) that featured moderately calcareous and alkaline soils. We found large genotype × environment interaction and modest or nil genetic correlation for line responses across locations for grain yield, a lime susceptibility score, and other traits, with the exception of individual seed weight and plant height. The GWAS identified significant SNP markers associated with various traits that were markedly inconsistent across locations, while providing direct or indirect evidence for widespread polygenic trait control. Genomic selection proved to be a feasible strategy, owing to a moderate predictive ability for yield and lime susceptibility in Larissa (the site featuring greater lime soil stress). Other supporting results for breeding programs where the identification of a candidate gene for lime tolerance and the high reliability of genome-enabled predictions for individual seed weight.

## 1. Introduction

Greater grain legume cultivation has a high priority for European agriculture, to enhance the sustainability of its agricultural systems [1,2], reduce its huge deficit for high-protein feedstuffs [3], and meet the increasing demand for healthy and nutritious plant-based foods [4]. White lupin (*Lupinus albus* L.) is a Mediterranean crop with a long history of domestication that has high potential interest in this context, because of its seed protein content close to 40% and other characteristics [5,6]. Its exploitation as a component of functional, healthy, or vegan food is favored by its good content of essential amino acids and several useful techno-functional properties [7], the ability of its γ-conglutin protein fraction to control glycaemia by interaction and binding with insulin and insulin-mimetic properties [8], and 8–12% seed content of oil with excellent nutritional characteristics [9]. While being less targeted by modern breeding than other cool-season grain legumes such as peas, faba beans, and narrow-leafed lupin, white lupin has out-performed these species in terms of crude protein yield per unit area across climatically-contrasting, autumn-sown rain-fed environments of southern Europe [10].

A key factor that limits the cultivation of white lupin worldwide is its narrow adaptation with respect to soil type. Grain and biomass reductions can be expected in soils whose active lime, i.e., the soluble fraction of calcium carbonate based on Drouineau’s [11] method, exceeds 1% [12], which are mildly calcareous, or in soils with an alkaline reaction [13]. Various studies have suggested that active lime has a greater depressive effect on crop yield than alkalinity [14,15,16], but these factors are associated in calcareous soils. Although Ca^2+^ may directly affect *Lupinus* species [17], the main effects of lime are indirect, through the precipitation of organic acids secreted by lupin cluster roots to mobilize and uptake phosphorus and iron [18], and through the inhibition of iron uptake by HCO_3_^−^ [13]. Indeed, lupin cluster roots increase in their relative proportion in response to limed soils, although the total root biomass decreases [19]. High soil Ca^2+^ concentration and alkaline soils also reduce the growth and the nodulating ability of the nitrogen-fixing *Bradyrhizobium* microsymbiont of lupins [13,20,21], but the selection of lime-tolerant plant material may have a greater impact on crop adaptation to calcareous soils than the exploitation of *Bradyrhizobium* strains adapted to such soils [22].

Various studies have highlighted the adaptation to moderately calcareous or alkaline soils of Egyptian landraces that evolved in such soils [23,24,25], but the exploitation of Egyptian genetic resources in breeding for autumn-sown environments of southern Europe is limited by their high susceptibility to winter low temperatures [26]. Variation in adaptation to moderately calcareous or alkaline soils was also found in commercial varieties and some accessions of indefinite origin [15], and within Italian landrace germplasm [22]. The phenotypic selection for white lupin lime tolerance may be complicated by high soil heterogeneity when performed under field conditions [27,28], and by abnormal plant root growth [29] and fairly modest correlation with genotype responses in agricultural environments [15] when performed in pots or liquid culture. In addition, lime tolerance and adaptation to other site-specific agroclimatic characteristics are confounded when assessing genotype yield responses under field conditions, suggesting to assess these responses across agroclimatically-contrasting sites that feature a calcareous soil. 

Plant breeding for traits that are genetically complex and/or difficult to assess could become more cost-efficient by genomic selection, which combines phenotyping and genotyping data of a genotype sample (training population) representing a target genetic base (reference population) into a statistical model for the prediction of breeding values in future plant selection [30,31]. This avenue has become practically feasible after the development of next generation sequencing techniques that genotype large germplasm sets by thousands of single nucleotide polymorphism (SNP) markers at a relatively low cost. Genotyping-by-sequencing (GBS; [32]), which skips sequence discovery and explores SNP polymorphism in DNA fragments cut by a restriction enzyme, holds special interest in this context [33]. One study confirmed the ability of GBS to generate thousands of polymorphic SNP markers for white lupin genetic analyses [34]. GBS-based, pioneer genomic selection studies were encouraging for white lupin breeding. The cross-environment predictive ability (as Pearson’s correlation between predicted and observed values) for grain yield between pairs of moderately contrasting environments, such as autumn-sown subcontinental vs. Mediterranean sites or moisture-favorable vs. drought-prone environments, was in the range of 0.40–0.51 [35]. Predictions were highly accurate (0.84–0.85) for the onset of flowering and winter survival, and moderately accurate (0.49–0.63) for various grain yield components and other morphophysiological traits, based on intra-environment cross-validations in a second study [36]. The practical interest of these findings for breeding programs was limited by the fact that they were based on relatively small genotypes sets (81 to 117 entries) mainly including landrace accessions.

This study focused on a collection of 140 GBS-genotyped sweet-seed breeding lines representative of a genetically-broad reference population originated by crosses of four elite landraces with four elite sweet-seed cultivars or breeding lines performed according to a factorial mating design. The lines were evaluated for grain yield and other traits associated with performance in an autumn-sown environment of Greece and a spring-sown environment of the Netherlands that featured moderately calcareous and alkaline soils. The main objectives of our study were to assess the phenotypic variation, the trait architecture based on a genome-wide association study (GWAS), and the predictive ability of genome-enabled models for grain yield and its contributing traits, verifying as well the consistency of genotype adaptation patterns and genome-based results across the two growing regions.

## 2. Results

### 2.1. Adaptive Responses

The inbred lines were evaluated under field conditions in Larissa (eastern continental Greece) and Ens (the Netherlands), two sites that featured moderately calcareous, moderately alkaline soils (Table 1). Actually, Larissa featured about 22% greater soil active lime than Ens, along with definitely lower rainfall over the crop cycle (Table 1). Site mean values of a nine-level lime susceptibility score recorded twice (at the end of the pod setting stage of the main stem and of the primary branches) indicated that plants underwent much greater stress in the Greek site (the score value of which corresponded to severe stress) than the Dutch site (the score value of which corresponded to mild stress) (Table 2). Accordingly, the Greek site exhibited distinctly lower mean values of crop yield, proportion of plants with seeds, plant height, and one component of the crop yield, namely, number of pods per plant (Table 2), compared with the Dutch location. Genetic variation among inbred lines was observed for all traits at both sites (*p* < 0.05), except for the proportion of plants with seeds, which was not significant in the Dutch site (in correspondence with a trait mean value approaching unity) (Table 2). The Greek location also showed greater broad-sense heritability for most traits that might be affected by susceptibility to soil lime (crop yield; lime susceptibility scores; proportion of plants with seeds; plant height), while showing heritability comparable to the Dutch site for the three component traits of crop yield (numbers of pods per plant and of seeds per pod; individual seed weight) (Table 2). 

Genotype × environment interaction occurred for all traits (*p* < 0.01; Table S1). It was quite large on the basis of the low or nil genetic correlation for line responses across locations that was observed for all traits, except plant height and individual seed weight (Table 2). In particular, the lines displayed just a modest genetic correlation for grain yield responses across sites (*r_g_* = 0.32, *p* < 0.10), while showing no genetic correlation for the last lime tolerance score or the average value of the two scores (Table 2). No genetic correlation emerged as well for one of the yield components, the number of seeds per pod. An additional analysis of variance in which the genotype factor was partitioned into the effects of the cross (16 crosses) and the line within cross indicated that GEI effects for all traits were mainly due to the interaction of the cross with the environment (Table S1). The consistently larger mean square values for the cross relative to the line within cross in the same analysis suggested the larger contribution of the former factor also to the variation for line mean values across environments for the different traits (Table S1).

**Figure 1:**
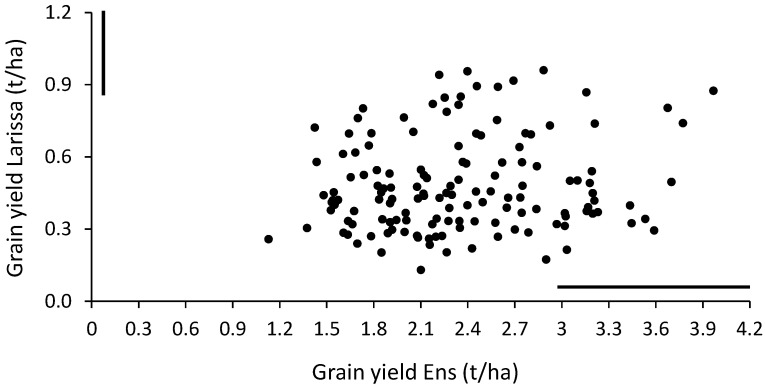
Grain yield of 140 white lupin inbred lines grown in Larissa (Greece) and Ens (the Netherlands). The vertical or horizontal bars indicate least-significant difference values at *p* < 0.05 for line comparison in the relevant site.

Yield responses of the lines in the two sites, which are graphically summarized in Figure 1, indicated the presence of a few lines characterized by good adaptation to both test sites. 

Line grain yield exhibited a high inverse phenotypic correlation with the lime susceptibility scores in the Greek site, and a moderate inverse correlation with these scores in the Dutch location (Table 3). This finding, indicating that the impact of lime tolerance on crop performance was stronger in the former site than the latter, was reinforced by the positive correlation of crop grain yield with plant height and the proportion of plants with seeds in the Greek site (Table 3). Number of pods per plant was the main grain yield component associated with crop yield at both sites (Table 3). The other yield components displayed inconsistent correlations with grain yield across sites; namely, a positive correlation for individual seed weight in Greece, and a positive correlation for the number of seeds per pod in the Dutch site (Table 3).

### 2.2. Genome-Wide Association Study

Genotyping-by-sequencing (GBS) of the DNA samples generated, on average, 1.93 million reads per sample. After alignment, SNP calling, and quality filtering, we obtained a set of 19,024 markers, which were further filtered for missing rate. We retained for molecular analyses 9815 polymorphic SNP markers mapped on the *Lupinus albus* genome released by Hufnagel et al. [37], after imposing the thresholds of 30% maximum missing rate per marker and 50% maximum missing rate per sample. For GS analyses, we also envisaged other thresholds of missing rate per marker, i.e., 15% and 20%, which produced 5429 and 6906 polymorphic SNP markers, respectively. 

On average, the linkage disequilibrium (LD) reached half of its 90th percentile (*r*^2^ = 0.28) at 3442 bp, with single chromosome values ranging from 277 bp for chromosome 12 to 6649 bp for chromosome 24 (Figure S1). We carried out an analysis of population structure by a discriminant principal components analysis (DPCA). Initially, the DPCA was performed to identify the optimal number of genotype groups (K) for imputation in GWAS and GS analyses by the k-means algorithm applied to increasing levels of K, selecting the value of K = 17 that minimized the Bayesian Information Content (BIC) (Figure S2, top panel). The final DPCA performed according to this K value, which retained seven components according to the a-score criterion, grouped the genotypes mainly on the ground of the 16 crosses they derived from. This is shown in Figure S2 (bottom panel), where genotype scores are reported in the space of the first two DPCA components. 

**Figure 2:**
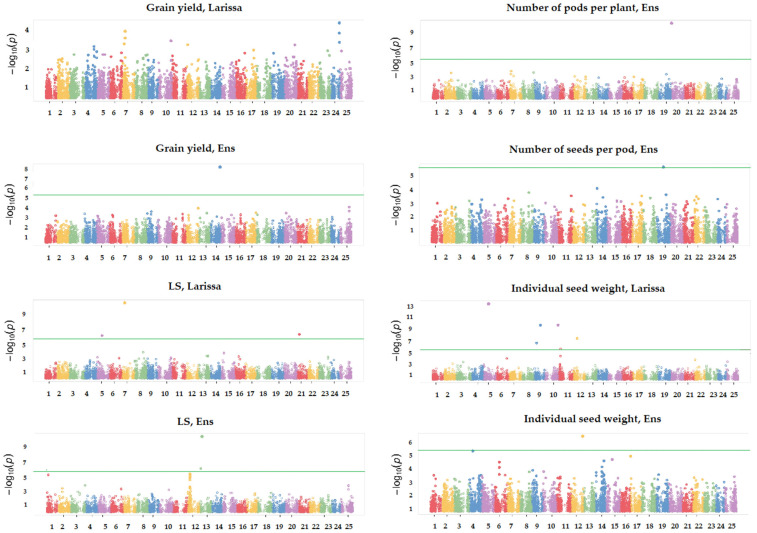
Manhattan plots showing the association scores of 9815 SNPs with grain yield, the average value of a visual lime susceptibility (LS) score, and two grain yield components featuring significant associations, for white lupin lines grown in Larissa (Greece) and Ens (The Netherlands). The continuous line represents Bonferroni’s threshold at *p* < 0.01.

**Figure 3:**
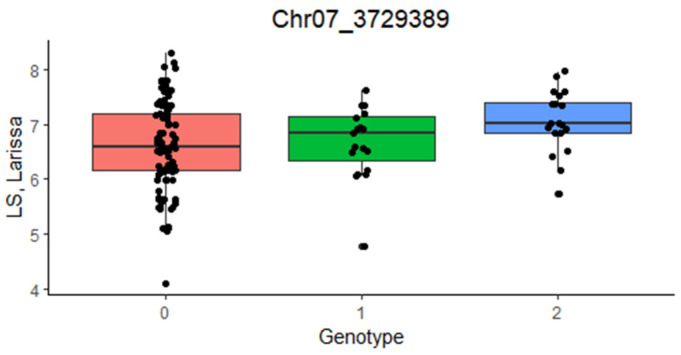
Boxplot showing the average value of a visual lime susceptibility (LS) score in Larissa (Greece) for the genotypic classes of the SNP Chr07_3729389, with 0 and 2 corresponding to the two homozygous classes and 1 to the heterozygous one. The thick black line represents the median, the box edges the 25th and 75th percentile, and the bar length the interquartile range.

The GWAS was performed on six traits that showed significant genetic variation in both locations, namely, grain yield, the mean value of the two lime susceptibility scores (preferred to the last score owing to its higher heritability in Larissa: Table 2), plant height, and the three grain yield component traits, with separate analyses for each site. Its main results are summarized by the Manhattan plots in Figure 2, which are limited to grain yield and to traits that displayed at least one significant SNP. The list of significant SNPs, and additional information on their minor allele frequency and estimated trait effect, are given in Table S2. For grain yield, no significant SNP emerged in Larissa, while one significant SNP was found on chromosome 14 in Ens. For the lime susceptibility score, we found three significant SNPs on the chromosomes 5, 7, and 21 in Larissa, and two significant SNPs on the chromosome 13 in Ens. However, one of the SNPs associated with lime susceptibility in the latter site contained no gene according to the lupin genome (Table S3), suggesting either a false positive or the presence of a regulatory region affecting the transcription of a relevant gene. In Larissa, the most-significant SNP for lime susceptibility, located on chromosome 7, corresponded to the second-ranking SNP for the association score of grain yield (which did not achieve *p* < 0.01 significance: Figure 2), reflecting to some extent the high phenotypic correlation between these traits in this site. This SNP was in high LD with a gene encoding a putative ferric-chelate reductase in the sequenced lupin genome (Lalb_Chr07g0184151; Table S3). The lime susceptibility score values of the genotypic classes in Figure 3 suggested an additive effect of this SNP, while the large within-class dispersion of values agreed with the expectation that other genomic regions besides the current one affect the white lupin lime tolerance.

For individual seed weight, we found six significant SNPs in Larissa (on chromosomes 5, 9, 10, 11, and 12) and one in Ens (also on chromosome 12, but not coincident with that in Larissa) (Figure 2). We also found one significant SNP for the number of pods per plant on chromosome 20 and one for the number of seeds per pod on chromosome 19 in Ens (Figure 2). No significant SNP emerged for these grain yield components in Larissa or for plant height in both locations (Figure S3). On the whole, the GWAS indicated no consistency between locations for significant SNPs. An additional GWAS performed on data averaged across locations of two traits that displayed high genetic correlation across locations, namely, individual seed weight and plant height (Table 2), revealed one significant SNP on chromosome 9 for the former trait, and no significant SNP for the latter trait (Figure S3).

### 2.3. Genome-Enabled Predictions

We assessed the intra-environment predictive ability of GS models as Pearson’s correlations between true and predicted phenotypes via ordinary cross-validation for the six traits that were the object of the GWAS. Table 4 reports for each trait the highest predictive ability obtained by the best combination of four tested statistical models, three thresholds of maximum missing rate per SNP marker (0.15, 0.20, 0.30), and the presence or absence of inputted population structure. We observed a consistently high predictive ability for individual seed weight (>0.6), a fairly low predictive ability for grain yield in Ens (<0.25), and a moderate predictive ability for all the remaining traits, including yield and the lime susceptibility score in Larissa (the site featuring greater lime soil stress). 

The list of best-performing GS models in Table 4 suggests that the inclusion of population structure mostly failed to improve the predictions, while there was no statistical model or missing rate threshold that tended to be consistently superior. The latter result was confirmed by intra-environment predictions reported for each GS model configuration in Figure S4, which showed modest variation as a function of the adopted model and missing rate threshold, and by the similar values of predictive ability averaged across traits that are reported in Table 5 for the four models on the one hand, and the three missing data thresholds on the other. A slight average advantage of the most stringent missing data threshold emerged, anyway, within each model in Table 5.

We assessed the cross-environment predictive ability (where one location was used to train the model for trait prediction in the other location) for the meaningful scenario represented by traits that displayed moderately high genetic correlation for line responses across locations, i.e., individual seed weight and plant height. Table 4 reports the predictive ability values averaged across the two possible training locations, given the modest differences observed as a function of the training site. As expected, the more challenging scenario represented by a cross-environment prediction determined a decrease in predictive ability compared with intra-environment prediction. However, the decrease was modest, i.e., 17% for plant height (0.331 vs. 0.400), and fairly modest, i.e., 27%, for seed weight (0.486 vs. 0.663), when comparing the mean value of cross-environment predictions with that of intra-environment predictions.

## 3. Discussion

The soil active lime content of the test sites was nearly (Ens) or over (Larissa) two-fold that indicated in [12] as the threshold for the occurrence of crop yield reduction due to soil lime stress. However, lime tolerance affected lupin genotype responses to a much greater extent in the Greek site than the Dutch site on the grounds of site mean values of the lime susceptibility scores and site-specific correlations of these scores with line grain yield, despite the only moderate difference between sites for soil active lime. Definitely greater drought stress caused by lower rainfall may have emphasized the negative effect of a calcareous soil in the Greek site, when considering that soil lime may determine a remarkable reduction of root biomass [19,29]. Better crop performance in the Dutch site may have been favored not only by the wetter climate, but also by greater soil N (Table 1) and more intense N fixation arising from seed inoculation, thereby partly compensating for the negative effect of soil lime.

An important finding of our study was the low consistency of genotype yield responses across the two sites featuring a moderately calcareous soil (as indicated by the *r_g_* value of 0.32). Various reasons may account for this result. First, the site-specific adaptive responses of the genotypes were not only influenced by lime susceptibility, but also by adaptation to the contrasting climates and sowing times of the two sites. Secondly, the much greater impact on plant responses of lime tolerance in the Greek site than in the Dutch site (as indicated by site mean values of the lime susceptibility score) could itself be a reason for GEI, making lime tolerance variation important mainly in the former site. In this context, data from the Greek site are more relevant than those from the Dutch site for assessing the lime tolerance of the lines, also with regard to lime tolerance score values, which suffered low heritability besides referring to milder stress in the Dutch site. Lime tolerance scores themselves displayed definite inconsistency between locations, suggesting that they might have estimated the general plant vigor (as determined by several possible site-specific factors) rather than the intrinsic lime tolerance, especially in the Dutch site (featuring lower soil lime stress). However, white lupin lime tolerance proved to be a fairly elusive and only partly repeatable trait in earlier studies. For example, 17 cultivars exhibited only moderate correlation (*r* = 0.50) for lime tolerance, expressed as shoot biomass ratio between high-soil lime and low-soil lime environments, across experiments in Italy and Morocco [22]; and the Egyptian landrace Giza 1 did not exhibit a distinct lime tolerance response in any of these experiments, while having emerged as lime tolerant in other studies [23,25]. The whole of these findings confirms the difficulty of improving the lupin adaptation to calcareous soils and suggests the importance of pursuing that on a regional basis, in order to also take account of climatic adaptation and its possible interaction with lime susceptibility. Selection for lime tolerance is also made challenging by the difficulty to highlight this trait by concurrent evaluation in high-soil lime and low-soil lime environments, because of the difficulty in identifying geographically-close locations with contrasting soil (as necessary to prevent the confounding of soil type effects with other environmental effects) and the limitations of evaluations performed in pots or hydroponic solutions (e.g., abnormal plant root growth [29]; modest reproduction of responses under field conditions [15]; lack of meaningful grain yield assessment). 

The number of pods per plant was the grain yield component that displayed the highest correlation with grain yield in both locations. This result agrees with results for landrace germplasm evaluated in Spanish environments [38,39]. Individual seed weight exhibited higher correlation with grain yield than the number of pods per plant or the number of seeds per pod in a multi-environment evaluation of a world germplasm collection in which seed weight featured greater genetic variation (based on CV_g_ values) than the other seed components [26], which was not the case here (Table 2). The current results, which are likely to be relevant for most breeding populations (whose variation for individual seed weight is unlikely to be very large), support the selection for more pods per plant whenever grain yield could not be meaningfully assessed, as in early selection stages.

The observed pattern of LD decay as a function of the physical distance was very similar to that reported for the same reference population of lines in an earlier study [40], with differences in the 90th percentile *r*^2^ and corresponding distance values essentially arising from the adoption of different window sizes for LD estimation (100 kb instead of 50 kb). However, the current LD decay was faster than that reported by Hufnagel et al. for a collection of cultivars and landraces [41], in the presence of substantial variation among chromosomes that was also found in that study. Although challenged by the fast LD decay and the somewhat suboptimal genotype sample size, our study revealed various quantitative trait loci (QTL) for crop yield and its component traits. However, these QTL were of region-specific interest, given their inconsistency across locations. This finding reflected the large GEI observed for most traits. The definite relationship between lime tolerance and crop yield that emerged phenotypically for the site featuring greater soil lime stress, i.e. Larissa, was confirmed genomically by the detection of a SNP on chromosome 7 that was associated with the former trait and tended to association with the latter trait. This SNP was in high LD with a gene encoding a putative ferric-chelate reductase (Lalb_Chr07g0184151), an enzyme that proved to affect lime tolerance in various other herbaceous [42,43] and tree species [44,45]. In particular, this enzyme participates in the absorption of iron, which is poorly available in soils featuring a high pH [46] and active lime [18], by reducing chelated Fe^3+^ according to the acquisition strategy I typical of non graminaceous species [47]. Our results support this gene as a strong candidate for improving white lupin tolerance to calcareous soils in future studies, albeit in the context of a polygenic control of lime tolerance that emerged from our study. 

The GWAS revealed very few or no significant SNPs for all traits in both sites, except for individual seed weight in Larissa, probably because of insufficient power to detect small-effect genes that are expected to be important for the control of crop yield, yield components, and plant stature. For individual seed weight, a polygenic trait architecture was supported not only by the detection of various putative QTL in one location, but by the fact that cross-environment GS prediction models exhibited just a modest loss in predictive ability (27%) compared with intra-environment prediction models despite the inconsistency for significant SNPs across locations. Such an outcome could only arise from the presence of many small-effect, statistically non-significant QTL that are consistent across locations.

Genomic selection is highly relevant for polygenic traits if predictions are proved to be sufficiently accurate. The current intra-environment predictive ability values for grain yield and the lime tolerance score in the site featuring greater soil lime stress (Larissa), which were close to 0.34, were lower than those reported for white lupin grain yield in moisture-favorable or drought stress conditions of landrace genotypes (in the range of 0.47–0.58) and breeding lines (in the range of 0.67–0.78) [35,40]. However, they could still justify the exploitation of the GS models for these traits for regional lime tolerance improvement (e.g., by concurrent selection according to genome-enabled predictions for both traits), when considering the challenges of phenotypic selection for lime tolerance. A second important indication for GS arising from this study is the high predictive ability of individual seed weight, which is supported not only by values for intra-environment prediction above 0.6, but also by the small loss of predictive ability when using a model constructed in one location for predictions in another, climatically-contrasting location. An earlier study on landrace accessions reported a similar intra-environment predictive ability value for this trait [36], further supporting the GS for this trait. A large seed (say, >0.50 g), which is needed for the traditional consumption of white lupin as a snack, is hardly found in modern cultivars [26]. The moderate predictive ability observed for plant height and the site-specific number of seeds per pod agrees with earlier findings for landrace germplasm [36].

The absence of sizable and consistent differences in predictive ability among GS statistical models is fairly common in general and was already reported in earlier white lupin studies that compared the same models [40] or a subset of them [35,36]. The slightly higher average predictive ability of GS models adopting the more stringent missing data threshold suggests that the 5429 polymorphic markers provided by this threshold were mostly sufficient for genome saturation aimed at GS prediction in this reference population.

In conclusion, this study highlighted the importance of GEI effects when improving white lupin adaptation to calcareous soils, supporting efforts on a regional scale that may take advantage of genome-enabled predictions of grain yield and visually-assessed lime tolerance. Future proof-of-concept work will verify the actual genetic gains obtained from GS for these traits applied to independent germplasm. Other major results issued from our study are: (a) the identification of a candidate gene for lime tolerance, revealed by the GWAS; (b) the high reliability across contrasting locations of the genome-enabled prediction of seed weight; and (c) the usefulness of more pods per plant as a phenotypic selection criterion in early selection stages that lack grain yield data.

## 4. Materials and Methods

### 4.1. Plant Material

The plant material for this study was represented by a sample of 140 sweet-seed inbred lines sorted from a reference population developed by CREA to broaden the genetic base for white lupin breeding in Europe. The reference population originated from 16 crosses produced by a 4 × 4 factorial mating design by which each of four elite sweet-seed cultivars or breeding lines was crossed with each of four elite bitter-seed, landrace accessions. The list of test lines and information on their parent germplasm is provided in Table S4. To further broaden the population genetic base, we used a different parent genotype within a landrace population for each cross with sweet-seed material (assuming that landraces are genetically heterogeneous, unlike modern cultivars). The landrace parent accessions were selected out of a world germplasm collection evaluated for grain yield under spring sowing in France and autumn sowing in two climatically-contrasting Italian sites [26]. Additional trait information for parent choice was provided from other studies relative to lime tolerance [22], genotype adaptation across Italian environments [48], and drought tolerance [49]. In brief, the Italian landrace LAP123 collected by CREA was selected because of moderately good adaptation to fairly calcareous soil; the landrace La646 from the Canary Islands, the Italian landrace La246 belonging to INRAE’s germplasm collection, the newly-registered Italian variety Arsenio (referred to as line 7–50 in earlier studies), and the French variety Lucky were selected because of wide adaptation to climatically-contrasting and/or moisture-contrasting environments; the Moroccan breeding line L27PS3 was chosen because of moderately high drought tolerance; the Greek landrace Gr56 (INRAE’s collection) and the breeding line MB-38 were selected because of high tolerance to low winter temperatures. Seed quality characteristics contributed to parent choice; e.g., the high γ-conglutin content of Arsenio and Lucky, or the very large seed of LAP123. The crosses were performed in 2014, whereas F_2_ to F_5_ inbred lines for each of the 16 crosses were produced from 2015 to 2017 by single-seed descent on-season and off-season generations. All crossing and seed multiplication work was carried out in isolation through insect-proof nets. Within-cross selection for low alkaloid content was performed (a) on F_3_ and F_4_ individual seeds by the fluorescence method [50], and (b) on F_4_ seed by a non-destructive test that was adapted to single seeds using the spectrophotometer method described in [51,52]. The latter test was used to discard material for which the alkaloid content value belonged to the highest 25% quartile. The final population included 960 F_5_ inbred lines (60 per cross), of which 560 (35 per cross) were genotyped, and 192 (12 per cross) were multiplied in isolation in 2018 to obtain the F_6_ seed used for this study and to phenologically characterize these lines. Four to 10 lines per cross contributed to the current set of 140 test lines (Table S4). The lines were randomly chosen within earlier-maturing crosses, and were selected for earliness within later-maturing crosses to avoid the presence in the panel of definitely winter-type germplasm (a phenological type expected to be poorly adapted to both target regions of this study). The number of lines issued by each individual parent was moderately balanced, ranging from 30 for MB-38 to 37 for La646, Arsenio, and L27PS3. 

### 4.2. Field Evaluation

The 140 inbred lines were evaluated under field conditions in Larissa (eastern continental Greece; 39°36′ N, 22°25′ E) and Ens (the Netherlands; 52°38′ N, 5°49′ E), two sites that featured moderately calcareous, moderately alkaline soils along with a contrasting climate (Table 1). In Larissa, characterized by a Mediterranean climate with mild winters and substantial terminal drought, the material was sown in late November 2020 and was combine-harvested in early July 2021. This site adopted a conventional management involving mineral fertilization with 46 kg/ha of P_2_O_5_, with manual weeding and no lupin seed inoculation. In Ens, characterized by a cool Oceanic climate, the material was sown at the beginning of April and was harvested by the end of October 2021. The crop was organically-managed, inoculating the seed by the commercial inoculant HiStick (Becker Underwood, Toulouse, France) before sowing, and performing occasional hand-weeding during the crop cycle. Each experiment was laid out according to a randomized complete block design with three replications per line, sowing 36 seeds per plot. Plots included 4 rows in Larissa and 3 rows in Ens, with rows at 25 cm spacing and seeds at 9 cm spacing on the row (density: 44.4 seeds/m^2^) in both sites. 

Grain yield at 0% seed moisture was assessed on a plot basis, after measuring the seed moisture on a sample of 100 seeds per plot. In Ens, the harvested pods were previously dried through a heated forced air drier for about 48 h. A lime tolerance score was visually attributed twice, i.e., at the end of the pod setting stage of the main stem, and at the end of the pod setting stage of the primary branches, according to the following scale definition: 1 (no stress): dark-green leaves, robust growth, 3–6 lateral branches, >20 pods/plant (only for the second observation); 3 (mild stress): chlorotic lower leaves, 2–4 lateral branches, 10–20 pods/plant (for the second observation); 5 (intermediate stress): yellow leaves along the whole plant, apparently limited shoot development, 1–2 lateral branches, 6–10 pods/plant (for the second observation); 7 (severe stress): yellow leaves along the whole plant, modest shoot development, abscission of leaves early in the ripening stage, 0–2 lateral branches, 2–5 pods/plant (for the second observation); 9 (extreme stress): yellow leaves along the whole plant, very poor shoot development, abscission of leaves and inflorescences, inability to complete the reproductive stage. Data analysis was performed for the latest score observation and for the average value of both observations. We also recorded the following traits: proportion of plants with seed; plant height at the end of flowering (measured on five test plants); average numbers of pods per plant and of seeds per pod (based on all test plants); and individual dry seed weight (assessed on 100 random seeds per plot). 

### 4.3. Statistical Analysis of Phenotyping Data

An analysis of variance (ANOVA) including the factors lupin genotype and block was performed for each trait in each environment to assess the significance of the variation among lines and its extent as a genetic coefficient of variation computed as:

CV = (*s_g_*/*m*) × 100 (1)

where *m* is the trait mean value, and *s_g_* is the square root of the genotypic component of variance (*s*^2^*_g_*) estimated along with the experimental error (*s*^2^*_e_*) component of variance by the REML method. Trait broad-sense heritability was computed from these components of variance by the equation:

*H*^2^ = *s*^2^*_g_*/(*s*^2^*_g_* + *s*^2^*_e_*/*n*) (2)

where *n* is the number of experiment replicates. We used *H^2^* values to compute best linear unbiased prediction (BLUP) values as described in [53], which were used for trait-marker analyses. Another ANOVA including the factors lupin genotype, environment, and block within environment was performed on each trait that displayed genetic variation in both sites, to test the significance of environmental and genotype × environment interaction (GEI) effects. The extent of the GEI effects was assessed by the size of the genetic correlation for lupin line responses across sites as described in [54] for one trait assessed in different environments. One last ANOVA including the fixed factors cross and environment and the random factors line within cross and block within environment partitioned the variation of genotype main effects and GEI into effects relative to the 16 crosses and the within-cross variability. The relationship of grain yield with the other traits was explored for each test site by phenotypic correlation. All analyses were carried out using SAS/STAT^®^ software 9.3 [55]. 

### 4.4. DNA Isolation, GBS Library Construction, and Sequencing

Genomic DNA was extracted from young leaves of F_5_ plants of each inbred line using the DNeasy Plant Mini Kit (Qiagen, Milan, Italy). Nucleic acid was quantified by a Quant-iT™ PicoGreen™ dsDNA Assay Kit (P7589, Life Technologies, Italy), checking its quality by 1% agarose gel electrophoresis. A trial digestion was carried out on 10% of the DNA samples using the Optizyme EcoRI restriction enzyme (25,000 U, Fisher BioReagents, Rodano, Italy), to compare bands of cut and uncut DNA. The reaction was performed at 37 °C for an hour and the enzyme was deactivated at 65 °C for 20 min. DNA samples were sent to The Elshire Group Ltd. lab (Palmerston North, New Zealand) for outsourced library preparation and sequencing. GBS data were generated according to Elshire et al.’s [32] method with the following changes: we used 100 ng of genomic DNA and 3.6 ng of total adapters, and restricted the genomic DNA with *Ape*KI enzyme (NEB New England Biolabs, R0643L, Ipswich, MA, USA); then, the library was amplified with Kapa Taq polymerase Alpha (KAPA Library Amplification Readymix, Kapa Biosystems KK2611, Cape Town, South Africa) by 14 PCR cycles. Sequencing was performed on a single Illumina HiSeq X lane, at 2 × 150 bp paired end. Adopting *Ape*KI as the restriction enzyme according to Elshire et al. [32] was supported by the fact that about 60% of the white lupin genome includes repetitive DNA sequences [37], which this enzyme tends to skip.

### 4.5. Genotype SNP Calling Procedures, Data Filtering, and Imputation

GBS raw reads were demultiplexed using Axe demultiplexer [56]. Trimming for restriction enzyme remnants, alignment on reference genome and SNP calling were performed using the dDocent pipeline [57]. For alignment, we used the *Lupinus albus* genome version 1.0 [37], which was downloaded from https://www.whitelupin.fr/ (accessed on 3 November 2022). The final genotype matrix, in the form of a vcf file, was further filtered for quality using the vcftools software [58] with parameters *−minQ 30 −max-non-ref-af 1 –non-ref-af 0.001*. The resulting data set was filtered for monomorphic markers, minor allele frequency (MAF) > 5%, missing SNP marker rate < 10%, 20% or 30%, and a missing rate per individual < 50%. Following Nazzicari et al. [59], we estimated missing data by random forest imputation [60] using the R package MissForest [61] with the configuration ntree = 100, maxiter = 10 and encoding genotypes as categorical data (factors).

Both genotypes and SNPs were filtered for excess heterozygosis by using the mean plus three and two standard deviations as maximum thresholds, respectively.

### 4.6. Analysis of Population Structure and Genome-Wide Association Study

The presence and pattern of population structure were investigated by a discriminant principal components analysis (DPCA) [62]. We iteratively used the k-means clustering algorithm for increasing values of K genotype groups from 1 to 20 to identify the optimal number of groups according to the local minimum of the Bayesian information criterion (BIC). The analyses were performed on the output of an ordinary principal components analysis performed on SNP data to benefit from its dimensionality reduction, but keeping all the components to avoid information loss. We performed the final DPCA after selecting the optimal K value. The optimal number of DPCA axis to retain for the following analyses was selected by the a-score criterion (which represents the propensity of DPCA toward overfitting). The whole procedure was implemented using the R package adegenet [63] using the functions *find.clusters(), dapc() and optim.a.score()*.

Linkage disequilibrium was estimated for SNPs within a 100 kb window in R by the LD.decay function from the package sommer [64]. We used genotype data filtered by 0.3 missing per marker, 0.5 missing per sample, 0.05 MAF, and excess SNP and genotype heterozygosis, plotted against physical distance, and fitted by a polynomial curve as described in [65]. Following data filtering, 136 genotypes were available for LD and GWAS analyses.

A GWAS was performed on BLUP data of grain yield, the average value across observations of the lime susceptibility score, plant height, number of pods per plant, number of seeds per pod, and individual seed weight measured in each location, using 9815 mapped SNPs according to the Blink model [66] in R package GAPIT3 [67]. A second GWAS was performed on data on plant height and individual seed weight averaged across locations. Each GWAS included the first seven components of the DPCA with K = 17, which appeared to properly account for population structure for all traits except individual seed weight in Ens, the only case for which the visual inspection of quantile-quantile plots comparing observed trait-marker association scores with those expected in case of no significant association suggested some inflation (Figure S5).

### 4.7. Genome-Enabled Predictions

For each of six focus traits, we tested several whole-genome regression models whose configuration resulted from the combination of four possible statistical models (described below), the presence or absence of population structure inputted as in the GWAS, and three thresholds of maximum missing rate per SNP marker (0.15, 0.20, and 0.30). We first envisaged an intra-environment prediction scenario, assessing the predictive ability of GS models by a standard within-location 10-fold cross-validation. Each analysis was performed 10 times, reporting the average result, to ensure numerical stability. We also envisaged a cross-environment prediction scenario for two traits that displayed moderately high genetic correlation for line responses across locations, using by turns one environment for training and the other for validation (by splitting the training data in a 90/10 fashion as done for intra-environment predictions). The whole procedure was repeated 10 times for numerical stability. 

We considered four possible whole-genome regression models: Ridge Regression BLUP (rrBLUP), Bayesian Lasso (BL), Bayesian Reproducing Kernel Hilbert space (RKHS), and Weighted G-BLUP (WGBLUP). All models were implemented using R package GROAN [68].

rrBLUP [69] assumes a linear mixed additive model where each marker is assigned an effect as a solution of the following equation:

y = 1μ + Wq + ε(3)

where y is the vector of observed phenotypes, μ is the mean of y, W is the genotype matrix (e.g., {0,1,2} for biallelic SNPs), q ∼ N (0, Iσ^2^_q_) is the vector of marker effects, and ε is the vector of residuals. The model is solved in a maximum likelihood context estimating the ridge parameter λ = σ^2^_e_/σ^2^_q_ representing the ratio between residual and markers variance. When the population structure is included in the model the above formula is updated as follows:

y = 1μ + Wq +Xb + ε (4)

where X is the is structure matrix with one row per sample and one column per considered DPCA component; and b ∼ N (0, Iσ^2^_b_) is the vector of (fixed) effects corresponding to the population structure.

BL [70] solves the same general model than rrBLUP but in the bayesian context where regression parameters have independent Laplace double-exponential priors. The system is solved via Gibbs sampling with proper iteration count (10,000 repetitions) and burn-in period (1000 repetitions) so as to ensure convergence. When present, the population structure was added to the model as a fixed (i.e., flat prior) component.

RKHS model is used to solve the so-called genomic BLUP (G-BLUP) in the Bayesian context. First, a genomic kinship additive matrix G is computed according to [71]. The matrix is then used in the following model:

y = 1μ + Zg +ε (5)

where Z is a design matrix allocating samples to genetic values and g is a vector of additive genetic effects for a sample with var(g) = Gσ^2^_g_, where G is the genomic relationship matrix and σ^2^_g_ is the genetic variance for this model. In the context of RKHS the G matrix is considered as the reproducing kernel function mapping from each pair of markers to covariance. The system is then solved with a standard Gibbs sampling, as done in BL, with the same configuration to accommodate for population structure if required.

WGBLUP [72] is very similar to RKHS and operatively follows the same implementation, with the main difference being that matrix G is substituted by matrix G*, which is computed by weighing the SNP markers by the *p* values resulting from an association study. The association scores were programmatically computed inside each cross-validation cycle on the training set using statgenGWAS R package [73]. Once the scores were obtained, the G* matrix was computed as:

G* = ZDZ′/[2Σp*_i_* (1 − p*_i_*)] (6)

where Z is an identity matrix for the markers, D is a diagonal matrix where each element of the diagonal corresponds to SNP weights, and p_i_ is the observed MAF of all genotyped individuals.

## Figures and Tables

**Table 1:** Soil variables and rainfall of two white lupin test locations.

Variable	Larissa (Greece)	Ens (The Netherlands)
Soil total CaCO_3_ content (g/kg) ^1^	61	50
Soil active CaCO_3_ content (g/kg) ^1,2^	22	18
Soil pH (in H_2_O) ^1^	7.6	7.9
Soil P_2_O_5_ (mg/kg) ^1^	11.0	5.2
Soil N (g/kg) ^1^	0.10	1.22
Soil texture class ^1^	Clay	Clay-loam
Rainfall over crop cycle (mm)	269	433

^1^ In the 0–40 cm soil layer. ^2^ As CaCO_3_ reacting to neutral NH_4_-oxalate.

**Table 2:** Mean value, genetic coefficient of variation (CV_g_), broad-sense heritability on a line mean basis (*H*^2^), *F* value for genotype × environment interaction (GEI), and GEI extent according to the genetic correlation for line responses across environments (*r_g_*), for grain yield, a visual lime susceptibility (LS) score, and five other traits recorded on 140 white lupin inbred lines grown in Larissa (Greece) and Ens (the Netherlands).

	Mean ^1^		CV_g_^2^		*H* ^2^			
Trait	Larissa	Ens	Larissa	Ens	Larissa	Ens	GEI *p* Value ^3^	*r_g_* ^4^
Dry grain yield (t/ha)	0.481 b	2.347 a	28.1 **	15.2 **	0.48	0.36	**	0.32 †
LS, mean of two scores (1–9)	6.65 a	2.53 b	13.3 **	16.4 **	0.77	0.39	**	0.03
LS, last score (1–9)	7.49 a	2.78 b	8.2 **	21.4 **	0.55	0.39	**	0.03
Proportion of plants with seeds	0.797 b	0.905 a	15.0 *	0.0 NS	0.64	0.00	**	−
Plant height (cm)	36.2 b	76.3 a	13.7 **	7.2 **	0.68	0.46	*	0.73 **
Number of pods per plant	2.63 b	9.23 a	19.0 **	16.0 **	0.43	0.48	**	0.33 †
Number of seeds per pod	2.56 b	2.76 a	20.8 **	13.7 **	0.57	0.58	**	−0.13
Individual seed weight (g)	0.253 a	0.248 a	18.7 **	11.9 **	0.72	0.71	**	0.68 **

^1^ Line means followed by different letter differ at *p* < 0.05. ^2^ NS, *, ** = variance between lines not different from zero and different from zero at *p* < 0.05 and *p* < 0.01, respectively. ^3^ *, ** = significant at *p* < 0.05 and *p* < 0.01, respectively; complete ANOVA results are reported in Table S1. ^4^ †, ** = different from zero at *p* < 0.10 and *p* < 0.05, respectively.

**Table 3:** Phenotypic correlation of grain yield with a visual lime susceptibility (LS) score and five other traits, for 140 white lupin inbred lines grown in Larissa (Greece) and Ens (the Netherlands).

Trait	Larissa	Ens
LS, mean of two scores	−0.68 **	−0.41 **
LS, last score	−0.72 **	−0.36 **
Proportion of plants with seeds	0.30 **	−
Plant height	0.52 **	0.00 NS
Number of pods per plant	0.63 **	0.41 **
Number of seeds per pod	−0.03 NS	0.36 **
Individual seed weight	0.32 **	0.01 NS

NS, ** = not different from zero and different from zero at *p* < 0.01, respectively.

**Table 4:** Predictive ability (as Pearson’s correlation between true and predicted phenotypes) of best-performing genomic selection models for intra-environment prediction (IP) of white lupin grain yield, the average value of a visual lime susceptibility (LS) score, three grain yield components and plant height observed in Larissa (GR, Greece) and Ens (NL, the Netherlands), and average value of best-performing models for cross-environment prediction (CP) of individual seed weight and plant height.

Trait [Prediction, Site]	Model ^1^	Population Structure Included	Maximum Missing Rate per SNP Marker	Predictive Ability ^2^
Dry grain yield [IP, GR]	rrBLUP	No	0.15	0.341
Dry grain yield [IP, NL]	RKHS	No	0.30	0.226
LS score [IP, GR]	rrBLUP	No	0.15	0.338
LS score [IP, NL]	RKHS	No	0.30	0.538
Number of pods per plant [IP, GR]	WGBLUP	No	0.15	0.275
Number of pods per plant [IP, NL]	RKHS	No	0.15	0.386
Number of seeds per pod [IP, GR]	WGBLUP	No	0.15	0.367
Number of seeds per pod [IP, NL]	WGBLUP	Yes	0.20	0.639
Individual seed weight [IP, GR]	WGBLUP	Yes	0.30	0.636
Individual seed weight [IP, NL]	RKHS	No	0.30	0.690
Plant height [IP, GR]	BL	Yes	0.15	0.387
Plant height [IP, NL]	RKHS	No	0.30	0.413
Individual seed weight [CP]	RKHS	No	0.15/0.20	0.486
Plant height [CP]	rrBLUP/BL	Yes	0.30	0.331

^1^ WGBLUP, Weighted G-BLUP; RKHS, Bayesian Reproducing Kernel Hilbert Space; BL, Bayesian Lasso. ^2^ The training procedure was repeated 10 times, reporting the mean values.

**Table 5:** Comparison of four statistical models for genomic selection based on their intra-environment predictive ability averaged across six traits listed in Table 4, for different thresholds of allowed missing data per SNP marker.

Model ^1^	Maximum Missing Rate per SNP Marker			
	0.15	0.20	0.30	Average
BL	0.411	0.402	0.400	0.405
RKHS	0.415	0.413	0.405	0.411
rrBLUP	0.416	0.412	0.407	0.412
WGBLUP	0.409	0.408	0.404	0.407
Average	0.413	0.409	0.404	0.409

^1^ BL, Bayesian Lasso; RKHS, Bayesian Reproducing Kernel Hilbert Space; Ridge Regression BLUP; WGBLUP, Weighted G-BLUP.

## Data Availability

Phenotypic data, genotypic data, square matrices of kinships and DPCA components for each of three thresholds of missing data per marker can be found in Data repository S1 available as Supplementary Material.

## Supplementary Materials

The following supporting information can be downloaded at: https://www.mdpi.com/article/10.3390/plants12051139/s1, Figure S1: LD decay plots for white lupin chromosomes based on Pearson’s correlation (*r*^2^) (Y axis) and physical distance (X axis) estimated on pairwise combinations of 9815 SNPs within a 100 kb window. Figure S2: Assessment of population structure by a discriminant principal components analysis (DPCA). Figure S3: Manhattan plots showing the association scores of 9815 SNPs with three white lupin traits observed in Larissa (Greece) or Ens (the Netherlands) that showed no significant association and two traits whose data were averaged across the two locations. Figure S4: Intra-environment predictive (as Pearson’s correlation between true and predicted phenotypes) of genomic selection models provided by the combination of four statistical models, three thresholds of allowed missing rate per SNP marker and the presence or absence of population structure, for white lupin grain yield, the average value of a visual lime susceptibility (LS) score, three grain yield components and plant height observed in Larissa (Greece) and Ens (the Netherlands). Figure S5: Quantile-quantile plots comparing the observed trait-marker association scores with those expected in case of no significant association for a GWAS based on 9815 SNPs performed for white lupin grain yield, the average value of a visual lime susceptibility (LS) score, three grain yield components and plant height observed in Larissa (Greece) and Ens (the Netherlands). Table S1: Analysis of variance (ANOVA) mean squares for grain yield, a visual lime susceptibility (LS) score, and five other traits recorded on 140 white lupin inbred lines issued from 16 crosses from a 4 × 4 factorial mating design which were grown in Larissa (Greece) and Ens (the Netherlands). The Line factor in the ANOVA (a) is subdivided into Cross and Line within Cross effects in the ANOVA (b). Table S2: Significant SNPs according to Bonferroni threshold at *p* < 0.01 detected by a GWAS based on 9815 SNPs, for white lupin grain yield, the average value of a visual lime susceptibility and three grain yield components observed in Larissa (Greece) and Ens (the Netherlands) or averaged across the two locations, with indication of minor allele frequency (MAF) and estimated trait effect. Table S3: List of genes potentially associated to the significant SNPs detected by a GWAS based on 9815 SNPs for white lupin grain yield, the average value of a visual lime susceptibility score and three grain yield components observed in Larissa (Greece) or Ens (the Netherlands) or averaged across the two locations, identified by scanning a region as long as the mean chromosome distance at which LD dropped to 0.2 in both directions from each significant SNP and reported with the relative annotated function (https://www.whitelupin.fr/, accessed on 15 December 2022). Table S4: List of 140 white lupin test inbred lines and their parent germplasm.

## Abbreviations

ANOVA	Analysis of variance
BL	Bayesian Lasso
RKHS	Bayesian Reproducing Kernel Hilbert Space
DPCA	Discriminant principal components analysis
GEI	Genotype × environment interaction
GBS	Genotyping-by-sequencing
GS	Genomic selection
LS	Lime susceptibility
QTL	Quantitative trait loci
REML	Restricted maximum likelihood
rrBLUP	Ridge Regression BLUP (best linear unbiased prediction)
SNP	Single nucleotide polymorphism
WGBLUP	Weighted G-BLUP
