# Supplementary material for: White Lupin Adaptation to Moderately Calcareous Soils: Phenotypic Variation and Genome-Enabled Prediction
Source: Plants (Basel). 2023 Mar 2;12(5):1139. doi: 10.3390/plants12051139 (PMC10005150; doi:10.3390/plants12051139)
Supplement: Supplementary file 1 [file plants-12-01139-s001.zip › supplementary Figure S1.pdf]

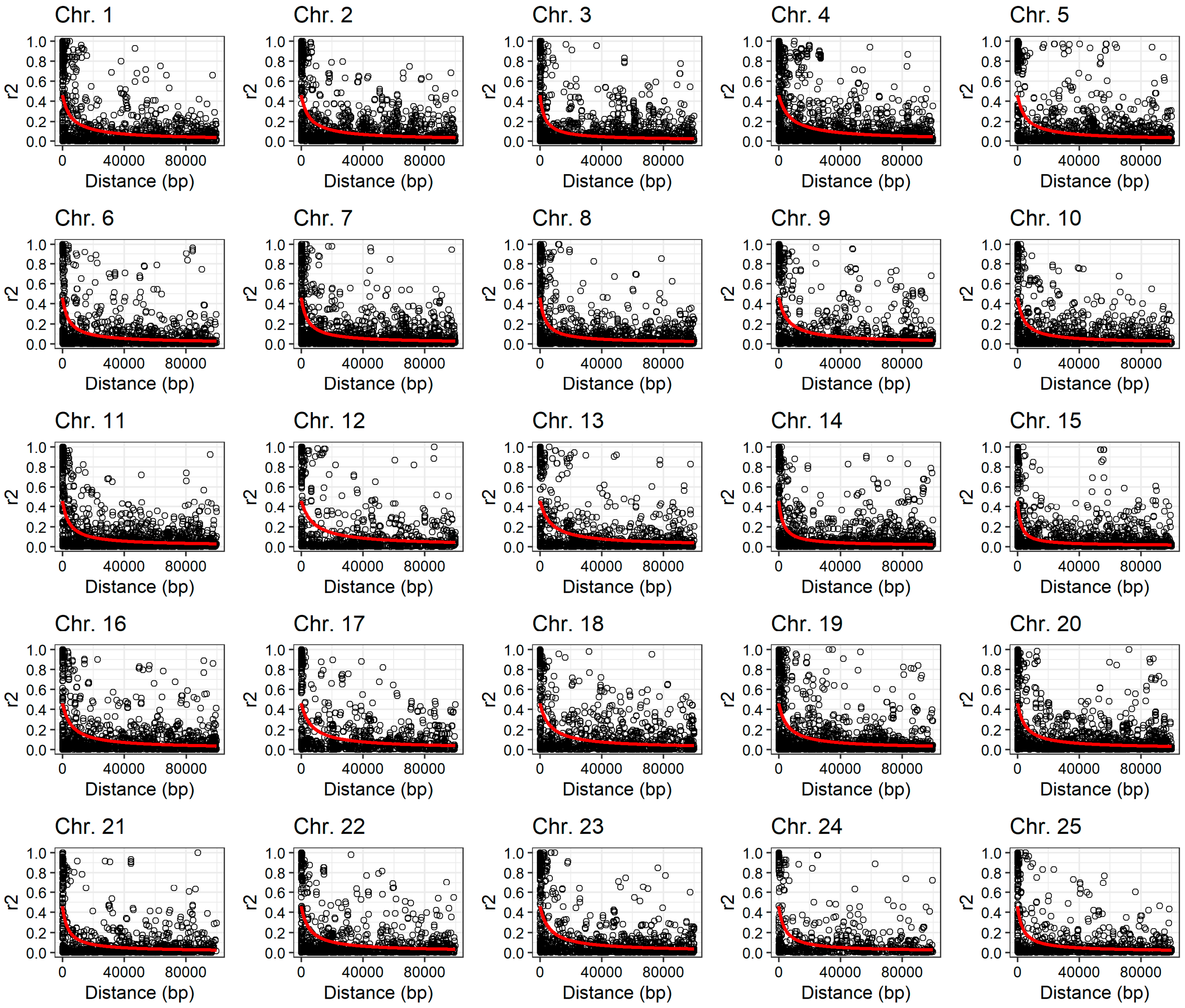

Supplementary Figure S1. LD decay plots for white lupin chromosomes based on Pearson's correlation (r^2) (Y axis) and physical distance (X axis) estimated on pairwise combinations of 9,815 SNPs within a 100 kb window.
