# Supplementary material for: White Lupin Adaptation to Moderately Calcareous Soils: Phenotypic Variation and Genome-Enabled Prediction
Source: Plants (Basel). 2023 Mar 2;12(5):1139. doi: 10.3390/plants12051139 (PMC10005150; doi:10.3390/plants12051139)
Supplement: Supplementary file 1 [file plants-12-01139-s001.zip › supplementary Figure S2.pdf]

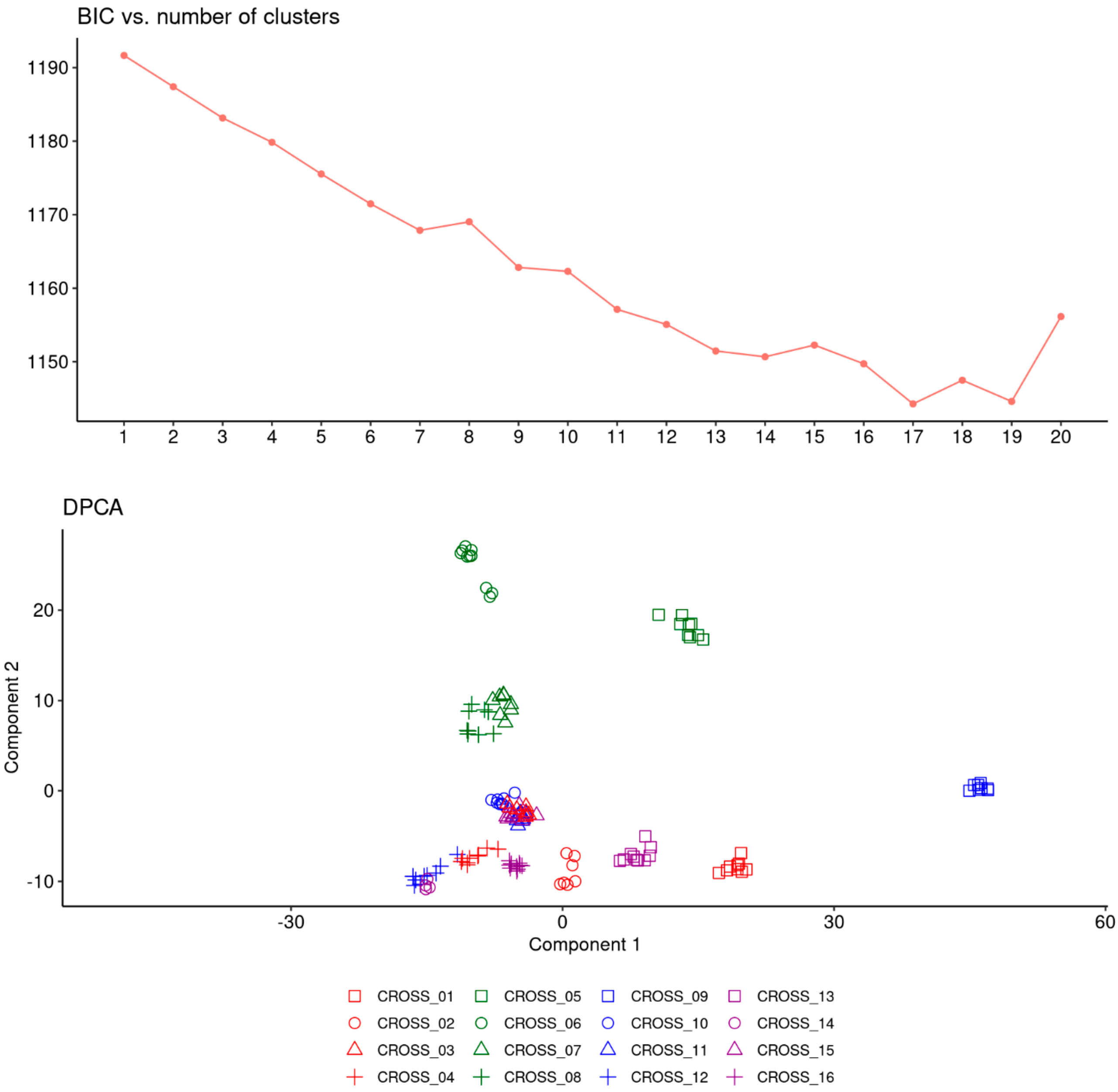

Supplementary Figure S2. Assessment of population structure by a discriminant principal components analysis (DPCA) analysis. The top panel shows the Bayesian Information Content (BIC) as measured after clustering the samples by the k-means algorithm for increasing levels of K. The bottom panel shows the first two DPCA components for the selected K level. The symbol shapes represent the sweet-seed parent line (Lucky: square; MB-38: circle; Arsenio: triangle; L27PS3: cross); the symbol colours represent the bitter-seed parent accession (Gr56: red; La646: green; La246: blue; LAP123: magenta).
