# Supplementary material for: White Lupin Adaptation to Moderately Calcareous Soils: Phenotypic Variation and Genome-Enabled Prediction
Source: Plants (Basel). 2023 Mar 2;12(5):1139. doi: 10.3390/plants12051139 (PMC10005150; doi:10.3390/plants12051139)
Supplement: Supplementary file 1 [file plants-12-01139-s001.zip › supplementary Figure S3.pdf]

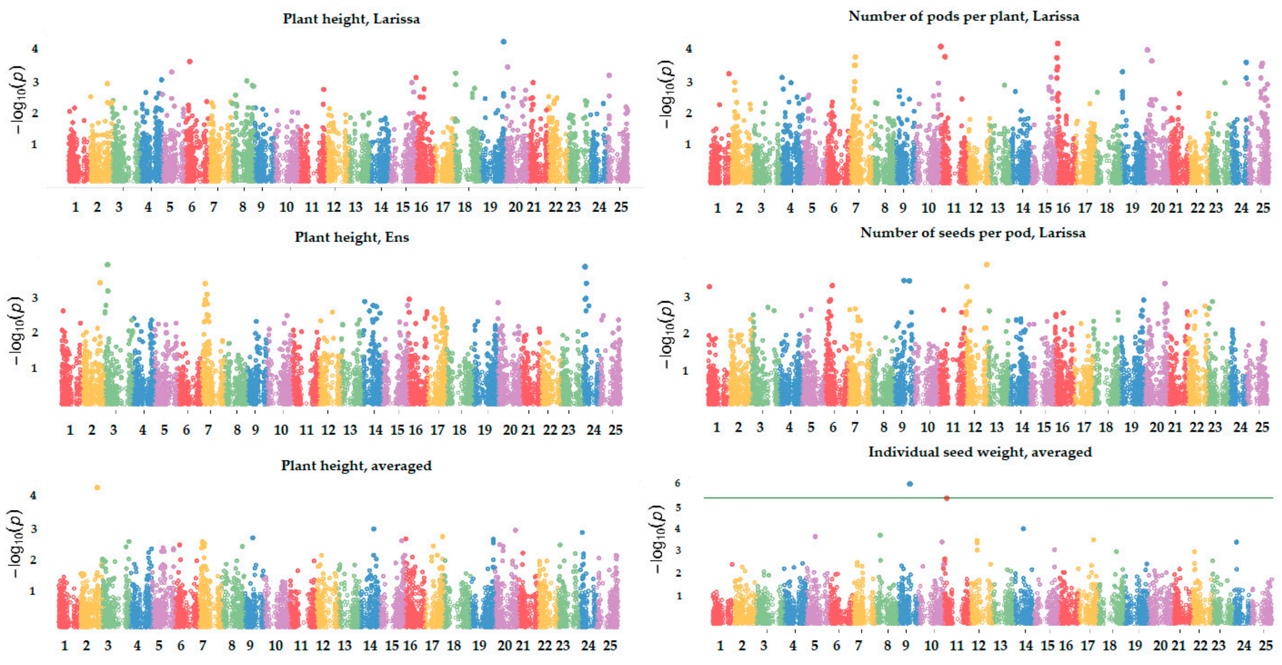

Supplementary Figure S3. Manhattan plots showing the association scores of 9,815 SNPs with three white lupin traits observed in Larissa (Greece) or Ens (the Netherlands) that showed no significant association and two traits whose data were averaged across the two locations. The continuous line represents Bonferroni threshold at $p < 0.01$.
