# Supplementary material for: White Lupin Adaptation to Moderately Calcareous Soils: Phenotypic Variation and Genome-Enabled Prediction
Source: Plants (Basel). 2023 Mar 2;12(5):1139. doi: 10.3390/plants12051139 (PMC10005150; doi:10.3390/plants12051139)
Supplement: Supplementary file 1 [file plants-12-01139-s001.zip › supplementary Figure S4.pdf]

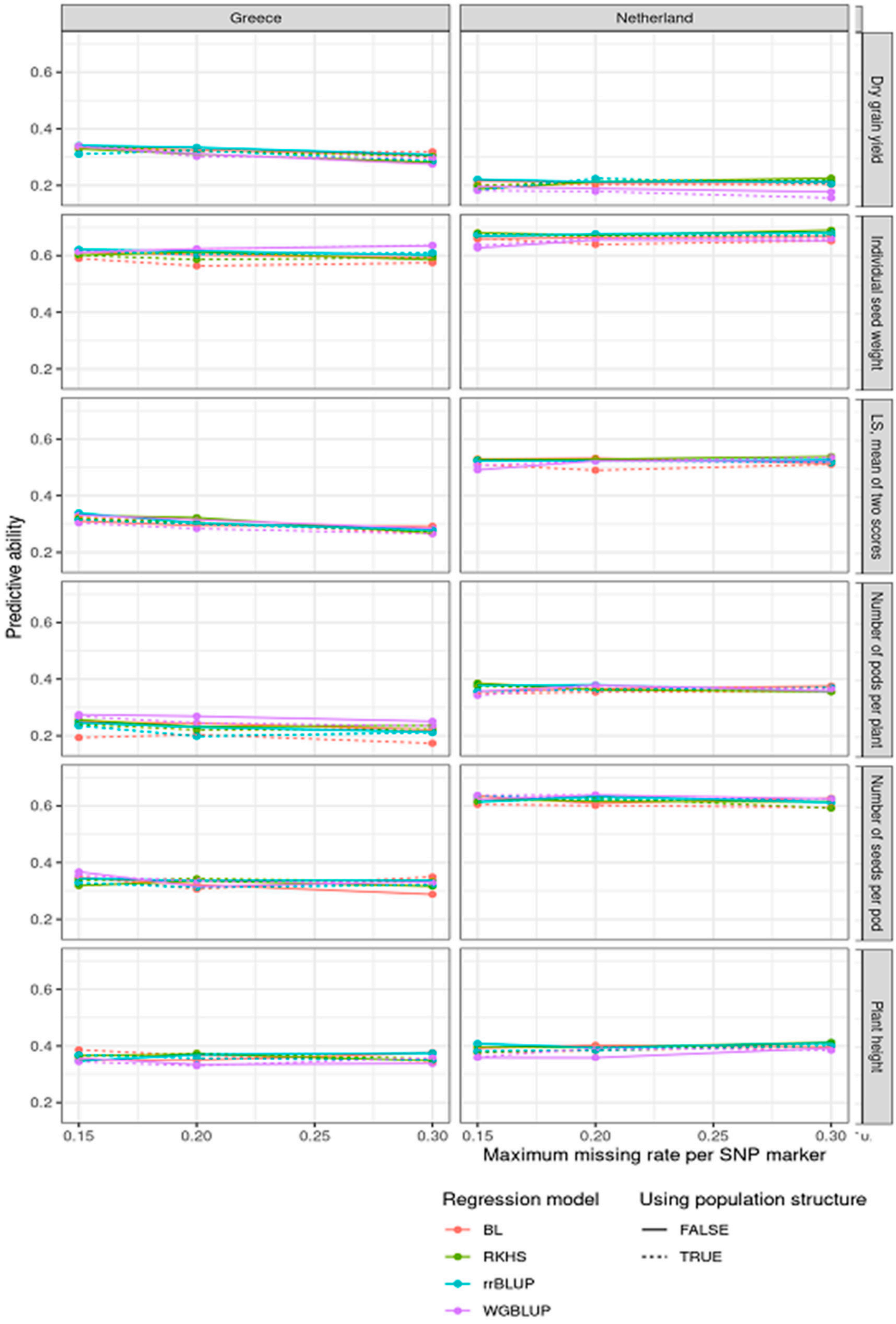

Supplementary Figure S4. Intra-environment predictive (as Pearson's correlation between true and predicted phenotypes) of genomic selection models provided by the combination of four statistical models, three thresholds of allowed missing rate per SNP marker and the presence or absence of population structure, for white lupin grain yield, the average value of a visual lime susceptibility (LS) score, three grain yield components and plant height observed in Larissa (Greece) and Ens (the Netherlands). BL, Bayesian Lasso; RKHS, Bayesian Reproducing Kernel Hilbert Space; Ridge Regression BLUP; WGBLUP, Weighted G-BLUP.
