# Supplementary material for: White Lupin Adaptation to Moderately Calcareous Soils: Phenotypic Variation and Genome-Enabled Prediction
Source: Plants (Basel). 2023 Mar 2;12(5):1139. doi: 10.3390/plants12051139 (PMC10005150; doi:10.3390/plants12051139)
Supplement: Supplementary file 1 [file plants-12-01139-s001.zip › supplementary Figure S5.pdf]

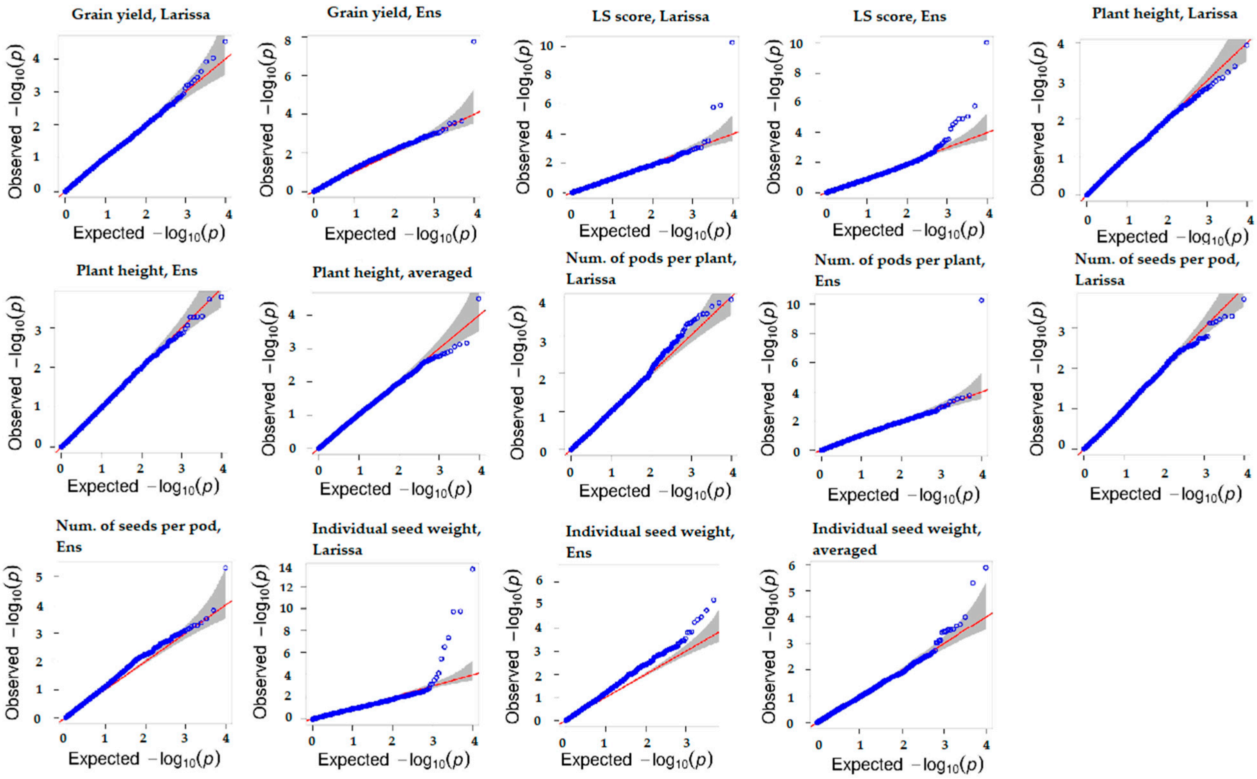

Supplementary Figure S5. Quantile-quantile plots comparing the observed trait-marker association scores with those expected in case of no significant association for a GWAS based on 9,815 SNPs performed for white lupin grain yield, the average value of a visual lime susceptibility (LS) score, three grain yield components and plant height observed in Larissa (Greece) and Ens (the Netherlands).
