# Supplementary material for: White Lupin Adaptation to Moderately Calcareous Soils: Phenotypic Variation and Genome-Enabled Prediction
Source: Plants (Basel). 2023 Mar 2;12(5):1139. doi: 10.3390/plants12051139 (PMC10005150; doi:10.3390/plants12051139)
Supplement: Supplementary file 1 [file plants-12-01139-s001.zip › supplementary Table S1.pdf]

Supplementary Table S1. Analysis of variance (ANOVA) mean squares for grain yield, a visual lime susceptibility (LS) score and five other traits recorded on 140 white lupin inbred lines issued from 16 crosses from a 4 × 4 factorial mating design which were grown in Larissa (Greece) and Ens (the Netherlands). The Line factor in the ANOVA (a) is subdivided into Cross and Line within Cross effects in the ANOVA (b).

Source of variation	Degrees of freedom	Dry grain yield	LS, mean of two scores	LS, last score	Proportion of plants with seed	Plant height	Number of pods per plant	Number of seeds per pod	Individual seed weight
ANOVA (a)									
Line	139	0.63 **	2.21 **	2.44 **	0.042 **	213.0 **	59.8 **	1.15 **	0.0093 **
Site	1	730.50 **	3561.85 **	4658.42 **	2.425 **	337425.0 **	140634.5 **	3627.80 **	0.0013 NS
Block (Site)	4	13.31	71.61	81.10	0.111	4462.7	295.6	3.08	0.0544
Line x Site	139	0.53 **	2.14 **	2.36 **	0.042 **	93.3 **	57.9 **	1.29 **	0.0036 **
Pooled error	556	0.36	0.75	1.30	0.020	70.4	31.5	0.50	0.0018
ANOVA (b)									
Cross	15	1.36 **	5.72 **	6.91 **	0.073 *	689.5 **	172.6 **	4.04 **	0.0484 **
Line (Cross)	124	0.53 **	1.78 **	1.90 **	0.038 **	155.4 **	46.5 **	0.61 **	0.0047 **
Site	1	715.54 **	3495.22 **	4568.70 **	2.220 **	329472.7 **	135673.3 **	2540.77 **	0.0000 NS
Block (Site)	4	13.31	71.61	81.10	0.111	4462.7	295.6	3.06	0.0544
Cross x Site	15	1.48 **	4.79 **	4.53 **	0.088 **	159.7 *	153.9 **	5.07 **	0.0079 **
Line (Cross) x Site	124	0.42 NS	1.82 **	2.10 **	0.036 **	85.2 NS	46.6 **	0.65 **	0.0031 **
Pooled error	556	0.36	0.75	1.30	0.020	70.4	31.5	0.50	0.0018

NS, *, ** = mean square value not significant and significant at $p < 0.05$ and $p < 0.01$, respectively.
