# Supplementary material for: White Lupin Adaptation to Moderately Calcareous Soils: Phenotypic Variation and Genome-Enabled Prediction
Source: Plants (Basel). 2023 Mar 2;12(5):1139. doi: 10.3390/plants12051139 (PMC10005150; doi:10.3390/plants12051139)
Supplement: Supplementary file 1 [file plants-12-01139-s001.zip › supplementary Table S2.pdf]

Supplementary Table S2. Significant ($p < 0.01$) SNPs detected by a GWAS based on 9,815 SNPs, for white lupin grain yield, the average value of a visual lime susceptibility score and three grain yield components observed in Larissa (Greece) or Ens (the Netherlands) or averaged across the two locations, with indication of *P* minor allele frequency (MAF) and estimated trait effect.

SNP	Trait	Location	MAF	Effect
Chr14_13626936	Dry grain yield (t/ha)	Ens	0.05	0.22
Chr07_3729389	Lime susceptibility (score 1-9)	Larissa	0.23	0.57
Chr21_3187412	Lime susceptibility (score 1-9)	Larissa	0.12	-0.42
Chr05_7315109	Lime susceptibility (score 1-9)	Larissa	0.11	-0.42
Chr13_2902405	Lime susceptibility (score 1-9)	Ens	0.06	0.25
Chr13_1073758	Lime susceptibility (score 1-9)	Ens	0.07	-0.16
Chr20_366132	Number of pods per plant	Ens	0.47	-0.74
Chr19_9007190	Number of seeds per pod	Ens	0.15	0.12
Chr05_6958697	Individual seed weight (g)	Larissa	0.08	0.032
Chr10_18335413	Individual seed weight (g)	Larissa	0.11	0.023
Chr09_8971510	Individual seed weight (g)	Larissa	0.19	-0.022
Chr12_5607461	Individual seed weight (g)	Larissa	0.10	-0.023
Chr09_3694527	Individual seed weight (g)	Larissa	0.20	0.014
Chr11_2644851	Individual seed weight (g)	Larissa	0.35	-0.014
Chr12_15889373	Individual seed weight (g)	Ens	0.42	-0.008
Chr09_8971510	Individual seed weight (g)	Averaged	0.19	-0.012
