# Supplementary material for: White Lupin Adaptation to Moderately Calcareous Soils: Phenotypic Variation and Genome-Enabled Prediction
Source: Plants (Basel). 2023 Mar 2;12(5):1139. doi: 10.3390/plants12051139 (PMC10005150; doi:10.3390/plants12051139)
Supplement: Supplementary file 1 [file plants-12-01139-s001.zip › supplementary Table S3.pdf]

Supplementary Table S3. List of genes potentially associated to the significant SNPs detected by a GWAS based on 9,815 SNPs for white lupin grain yield, the average value of a visual lime susceptibility score and three grain yield components observed in Larissa (Greece) or Ens (the Netherlands) or averaged across the two locations, identified by scanning a region as long as the mean chromosome distance at which LD dropped to 0.2 in both directions from each significant SNP and reported with the relative annotated function (https://www.whitelupin.fr/).

SNP	Scanned Interval	Trait	Location	Gene	Function
Chr14_13626936	Lalb_Chr14:13623014..13630858	Grain yield	Ens	Lalb_Chr14g0374491	Putative adenosinetriphosphatase
Chr14_13626936	Lalb_Chr14:13623014..13630858	Grain yield	Ens	Lalb_Chr14g0374501	Putative sulfate-transporting ATPase
Chr07_3729389	Lalb_Chr07:3723419..3735359	LS score	Larissa	Lalb_Chr07g0184141	Putative diacylglycerol kinase (ATP)
Chr07_3729389	Lalb_Chr07:3723419..3735359	LS score	Larissa	Lalb_Chr07g0184151	Putative ferric-chelate reductase (NADH)
Chr21_3187412	Lalb_Chr21:3182314..3192510	LS score	Larissa	Lalb_Chr21g0309921	Uncharacterized conserved protein
Chr21_3187412	Lalb_Chr21:3182314..3192510	LS score	Larissa	Lalb_Chr21g0309931	Putative RNA helicase transcription factor interactor and regulator CCHC(Zn) family
Chr05_7315109	Lalb_Chr05:7307410..7322808	LS score	Larissa	Lalb_Chr05g0223311	Putative glucan endo-1,3-beta-D-glucosidase
Chr05_7315109	Lalb_Chr05:7307410..7322808	LS score	Larissa	Lalb_Chr05g0223331	Profilin-1
Chr13_2902405	Lalb_Chr13:2892911..2911899	LS score	Ens	NA	NA
Chr13_1073758	Lalb_Chr13:1064264..1083252	LS score	Ens	Lalb_Chr13g0291101	Putative peptidase S1, PA clan

Chr13_1073758	Lalb_Chr13:1064264..1083252	LS score	Ens	Lalb_Chr13g0291111	Putative tetratricopeptide-like helical domain, pentacotripeptide-repeat region of PRORP
Chr13_1073758	Lalb_Chr13:1064264..1083252	LS score	Ens	Lalb_Chr13g0291121	Putative ribosomal protein L13
Chr13_1073758	Lalb_Chr13:1064264..1083252	LS score	Ens	Lalb_Chr13g0291131	Putative RNA-directed DNA polymerase
Chr20_366132	Lalb_Chr20:358851..373413	Number of pods per plant	Ens	Lalb_Chr20g0108311	Putative ATP citrate synthase
Chr20_366132	Lalb_Chr20:358851..373413	Number of pods per plant	Ens	Lalb_Chr20g0108321	Putative membrane transport protein
Chr20_366132	Lalb_Chr20:358851..373413	Number of pods per plant	Ens	Lalb_Chr20g0108331	Putative membrane transport protein
Chr19_9007190	Lalb_Chr19:8999259..9015121	Number of seeds per pod	Ens	Lalb_Chr19g0128161	Putative mitotic-spindle organizing protein
Chr19_9007190	Lalb_Chr19:8999259..9015121	Number of seeds per pod	Ens	Lalb_Chr19g0128171	Putative S-adenosyl-L-methionine-dependent methyltransferase
Chr09_8971510	Lalb_Chr09:8963033..8979987	Individual seed weight	Larissa; averaged	Lalb_Chr09g0332501	Putative CDP-diacylglycerol--inositol 3-phosphatidyltransferase, Long-chain-fatty-acid--CoA ligase
Chr09_8971510	Lalb_Chr09:8963033..8979987	Individual seed weight	Larissa; averaged	Lalb_Chr09g0332511	Putative nucleic acid-binding protein
Chr05_6958697	Lalb_Chr05:6950998..6966396	Individual seed weight	Larissa	Lalb_Chr05g0222881	Putative protein kinase RLK-Pelle-PERK-1 family

Chr05_6958697	Lalb_Chr05:6950998..6966396	Individual seed weight	Larissa	Lalb_Chr05g0222891	putative protein
Chr10_18335413	Lalb_Chr10:18329549..18341277	Individual seed weight	Larissa	Lalb_Chr10g0106121	Putative proteasome component Ecm29
Chr10_18335413	Lalb_Chr10:18329549..18341277	Individual seed weight	Larissa	Lalb_Chr10g0106131	putative protein
Chr10_18335413	Lalb_Chr10:18329549..18341277	Individual seed weight	Larissa	Lalb_Chr10g0106141	hypothetical protein
Chr10_18335413	Lalb_Chr10:18329549..18341277	Individual seed weight	Larissa	Lalb_Chr10g0106151	Putative F-box domain-containing protein
Chr12_5607461	Lalb_Chr12:5596474..5618448	Individual seed weight	Larissa	Lalb_Chr12g0204671	Putative tetratricopeptide-like helical domain, DYW domain-containing protein
Chr12_5607461	Lalb_Chr12:5596474..5618448	Individual seed weight	Larissa	Lalb_Chr12g0204681	hypothetical protein
Chr12_5607461	Lalb_Chr12:5596474..5618448	Individual seed weight	Larissa	Lalb_Chr12g0204691	Putative T-complex protein 1, alpha subunit
Chr12_5607461	Lalb_Chr12:5596474..5618448	Individual seed weight	Larissa	Lalb_Chr12g0204701	Putative rossmann-like alpha/beta/alpha sandwich protein
Chr12_5607461	Lalb_Chr12:5596474..5618448	Individual seed weight	Larissa	Lalb_Chr12g0204711	Putative ADF/Cofilin, ADF-H/Gelsolin-like domain-containing protein
Chr09_3694527	Lalb_Chr09:3686050..3703004	Individual seed weight	Larissa	Lalb_Chr09g0325971	Putative Ecd family protein

Chr09_3694527	Lalb_Chr09:3686050..3703004	Individual seed weight	Larissa	Lalb_Chr09g0325981	hypothetical protein
Chr09_3694527	Lalb_Chr09:3686050..3703004	Individual seed weight	Larissa	Lalb_Chr09g0325991	Putative mechanosensitive ion channel MscS, LSM domain-containing protein
Chr11_2644851	Lalb_Chr11:2638652..2651050	Individual seed weight	Larissa	Lalb_Chr11g0065601	Putative peptidase S54, rhomboid
Chr11_2644851	Lalb_Chr11:2638652..2651050	Individual seed weight	Larissa	Lalb_Chr11g0065611	Putative peptidase S54, rhomboid
Chr12_15889373	Lalb_Chr12:15878386..15900360	Individual seed weight	Ens	Lalb_Chr12g0208881	Putative X8 domain-containing protein
Chr12_15889373	Lalb_Chr12:15878386..15900360	Individual seed weight	Ens	Lalb_Chr12g0208891	putative protein
